# Rapid Heme Transfer Reactions between NEAr Transporter Domains of *Staphylococcus aureus*: A Theoretical Study Using QM/MM and MD Simulations

**DOI:** 10.1371/journal.pone.0145125

**Published:** 2015-12-14

**Authors:** Yoshitaka Moriwaki, Tohru Terada, Kouhei Tsumoto, Kentaro Shimizu

**Affiliations:** 1 Department of Biotechnology, Graduate School of Agricultural and Life Sciences, The University of Tokyo, Tokyo, Japan; 2 Agricultural Bioinformatics Research Unit, Graduate School of Agricultural and Life Sciences, The University of Tokyo, Tokyo, Japan; 3 Department of Chemistry and Biotechnology, Graduate School of Engineering, The University of Tokyo, Tokyo, Japan; Montana State University, UNITED STATES

## Abstract

In vertebrates, most iron is present as heme or is chelated by proteins. Thus, Gram-positive pathogens such as *Staphylococcus aureus* have evolved an iron-regulated surface determinant (Isd) system that transports heme across thick cell walls into the cytoplasm. Recent studies have demonstrated that heme is rapidly transferred between the NEAr Transporter (NEAT) domains of the Isd system, despite its high affinity toward each domain, suggesting the presence of an intermediate NEAT•heme•NEAT complex. In the present study, we performed short restrained molecular dynamics (MD) simulations to dock the acceptor NEAT domain to the donor NEAT•heme complex and obtained models where the two NEAT domains were arranged with two-fold pseudo symmetry around the heme molecule. After turning off the restraints, complex structures were stably maintained during subsequent unrestrained MD simulations, except for the hydrogen bond between the propionate group of the heme molecule and the donor NEAT domain, potentially facilitating the transition of heme from the donor to the acceptor. Subsequent structural optimization using the quantum mechanics/molecular mechanics (QM/MM) method showed that two tyrosine residues, one from each NEAT domain, were simultaneously coordinated to the ferric heme iron in the intermediate complex only if they were deprotonated. Based on these results, we propose a reaction scheme for heme transfer between NEAT domains.

## Introduction

Iron is ubiquitous in biological systems and plays various roles in the growth and activity of all living organisms. Bioavailable iron is predominantly incorporated into protoporphyrin structures such as heme, which play active roles in respiration as cofactors of cytochromes and in electron transport between various proteins. Because hemoglobin is the most abundant hemoprotein in vertebrates, pathogenic bacteria have evolved various molecular mechanisms to separate and sequester heme from hemoglobin. These mechanisms involve the transfer and degradation of heme and subsequent extraction of the iron atom.

X-ray crystallographic studies have elucidated the molecular bases of protein functions involved in bacterial heme uptake. Although heme transfer mechanisms differ between Gram-negative and Gram-positive bacteria, mechanisms of heme import and metabolism are generally similar. In particular, Gram-negative bacteria are encapsulated in a <10-nm-thick peptidoglycan layer [1–3] and an outer membrane. The extracellular hemophore protein HasA was first identified in Gram-negative *Serratia marcescens* [4, 5] as a protein that sequesters and delivers heme from host hemoproteins such as hemoglobin to the outer membrane receptor HasR [6]. HasA binds HasR with high affinity (*K*_*d*_ = 5 nM), regardless of its heme-loaded status [7], and the mechanisms of heme transfer between these proteins have been characterized in crystallographic studies of the HasA–HasR complex [8]. These analyses indicate that binding of HasR to HasA decreases the affinity of heme toward HasA, leading to dissociation, diffusion, and subsequent binding to HasR [8]. Heme is then imported into the cytosol by the TonB•ExbB•ExbD inner membrane complex and an ATP transporter [9]. In contrast with Gram-negative bacteria, Gram-positive pathogens such as *Staphylococcus aureus* and *Bacillus anthracis* have thick (20–80 nm [10]) peptidoglycan cell walls and lack outer membranes. Thus, heme transfer into *S*. *aureus* requires the expression of the iron-regulated surface determinant (Isd) proteins IsdH, IsdB, IsdA, and IsdC. These proteins are anchored to the cell wall and have one or more copies of the conserved NEAr Transporter (NEAT) domain, which binds hemoglobin and performs heme transfer. Recent studies on IsdB have shown that its N-terminal segment, the hemoglobin-binding NEAT domain (IsdB-NEAT1), and the linker domain concertedly contribute to a direct transfer of heme from hemoglobin to the heme-binding NEAT domain (IsdB-NEAT2) [11–13]. It is also expected that IsdH-NEAT1 and -NEAT2 domains bind hemoglobin to extract heme and the NEAT3 domain receive it in a similar manner. Heme is subsequently transferred across the cell wall by IsdA-NEAT (IsdA-N) and IsdC-NEAT (IsdC-N) toward the membrane lipoprotein IsdE [14–16] (also see Fig A in S1 File).

**Fig 1:**
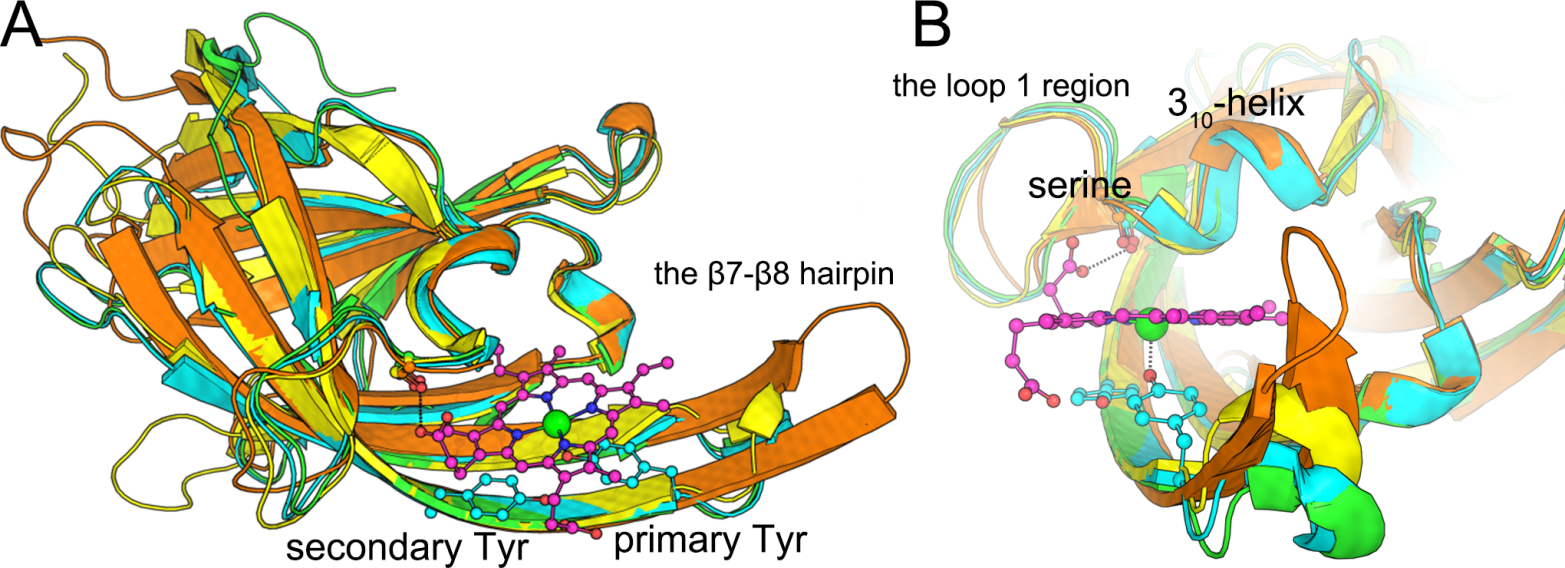
Superposition of IsdH-N3 (cyan), IsdB-N2 (green), IsdA-N (yellow), and IsdC (orange) crystal structures. (A) Overview of Isd–NEAT domains; the heme molecule and primary/secondary tyrosine residues on the β8 strand are represented as ball-and-stick models; the iron atom of heme is shown as a green sphere. (B) Close-up view of the heme-binding pocket; heme propionate groups form H-bonds with conserved serine residues between the loop 1 region and the 3_10_-helix (IsdH–Ser563, IsdB–Ser361, IsdA–Ser82, or IsdC–Ser47).

IsdH-N3 [17], IsdB-N2 [18], IsdA-N [19], and IsdC-N [20, 21] have very high structural similarity (RMSD < 2 Å), despite having low sequence identity (about 20%; Fig 1A). Moreover, they share a conserved YXXXY motif on β8 and a conserved serine residue on the 3_10_-helix. Hence, we refer to N-terminal (IsdH–Tyr642, IsdB–Tyr440, IsdA–Tyr166, and IsdC–Tyr132) and C-terminal tyrosine residues (IsdH–Tyr646, IsdB–Tyr444, IsdA–Tyr170, and IsdC–Tyr136) in the YXXXY motif as primary and secondary tyrosine residues, respectively. The primary tyrosine directly coordinates heme iron, whereas the secondary tyrosine forms a hydrogen bond with the primary tyrosine. In contrast with most hemoproteins, one side of the heme molecule in complexes with Isd proteins is almost exposed to solvent (Fig 1).

IsdH-N3, IsdA-N, and IsdC-N bind heme with high affinity (*K*_*d*_ = 6.5–34 nM), and downstream domains produce higher affinity binding than upstream domains [22]. In our previous study, we showed that differences in amino acids in the loop 1 region (between the β1b strand and the 3_10_-helix) and the salt bridge between Glu88 and Arg100 are peculiar to IsdC and are responsible for differences in heme binding affinity [22]. Subsequently, we demonstrated that heme is transferred according to the thermodynamic equilibrium principle [22].

Despite high affinity binding to NEAT domains, heme is efficiently transferred to downstream domains [14, 23, 24], with a transfer rate between holo-IsdA and apo-IsdC of 54.3 ± 1.8 s^−1^ [14]. This rate is approximately 70,000 times greater than the dissociation rate of heme (0.00076 s^−1^) [14], suggesting the presence of a protein–protein intermediate complex. Two “handclasp models” have been proposed for the structure of this complex. In particular, a model derived from nuclear magnetic resonance paramagnetic relaxation enhancement experiments suggested loose binding of the two domains and movement of heme approximately 18 Å from the donor domain to the acceptor domain, as in the HasA–HasR complex [23]. However, in the other model, the two domains are bound tightly, and the primary tyrosine residues from donor and acceptor proteins are simultaneously coordinated with heme iron [25, 26]. This latter “tight handclasp model” may be superior to the former model in terms of the stability of the complex and the efficiency of heme transfer. However, the validity of this model, particularly with reference to the coordination structure of the heme iron with two axial tyrosine ligands, has not yet been established.

In the present study, we initially assessed the validity of this coordination structure using quantum mechanics (QM) calculations. Subsequently, we generated a model of the intermediate complex structure using classical molecular dynamics (MD) simulations. The model was then further refined using a hybrid quantum mechanics/molecular mechanics (QM/MM) method, and a mechanism for heme transfer was proposed.

## Methods

### QM calculations of the Fe(III)-porphine-(phenolate)_2_ complex

The initial coordinates of Fe(III)-porphine (Fe^3+^-C_20_H_12_N_4_) and a phenolate anion were taken from the corresponding parts of heme and the primary tyrosine residue of the crystal structure of IsdH-N3 (Protein Data Bank [PDB] ID, 2Z6F) [17], respectively. Relative to the iron atom, the second phenolate was positioned symmetrically with the first, and the two phenolate molecules were placed at axial positions accordingly (see also Fig B in S1 File). Based on electrospray ionization mass spectrometry (ESI-MS) and magnetic circular dichroism (MCD) studies of the NEAT•heme complexes [24, 27–30], the heme iron atom was assumed to be in a ferric high-spin state. Accordingly, a relaxed potential energy surface (PES) scan with the two Fe–O bonds was performed using Gaussian 09 software [31] at the B3LYP level [32, 33] with the 6-31G(d) basis set for H, C, N, and O atoms and the LANL2DZ effective core potential (ECP) basis set [34] for the iron atom.

### MD simulations

In the present study, two intermediate complex models (IsdH-N3•heme•IsdA-N and IsdA-N•heme•IsdC-N) were generated using restrained MD simulations starting from the initial arrangements, in which the two NEAT domains are separated. To generate the initial structures, we constructed tentative models of ternary complexes with heme sandwiched between two NEAT domains. In these models, four N atoms of heme bound to one NEAT domain were superimposed on those bound to the other NEAT domain, and one heme molecule was then removed. The coordinates of IsdH-N3•heme, IsdA-N•heme, and IsdC-N•heme were obtained from PDB (IDs, 2Z6F [17], 2ITF [19], and 2O6P [20], respectively), and the two domains of the tentative complex model were separated from each other. As for the IsdH-N3•heme•IsdA-N system, IsdA-N was translated by 20 Å parallel to the vector from the IsdH–Tyr642 Cα atom to the IsdH–Tyr646 Cα atom and by 10 Å perpendicular to the heme plane (Fig C in S1 File). Similarly, in the IsdA-N•heme•IsdC-N system, IsdC-N was moved by 30 Å parallel to the vector from the IsdA–Tyr166 Cα atom to the IsdA–Tyr170 Cα atom and by 10 Å perpendicular to the heme plane (Fig C in S1 File). In both systems, N- and C-termini were capped with acetyl and *N*-methyl groups, respectively. Protonation states of histidine residues were determined using PROPKA3 (pH 7.0) [35]. The primary tyrosine residues IsdH–Tyr642, IsdA–Tyr166, and IsdC–Tyr132 were deprotonated. The force field parameters for heme and the primary tyrosine residues of the donor proteins (IsdH-N3 in the IsdH-N3•heme•IsdA-N system and IsdA-N in the IsdA-N•heme•IsdC-N system) that coordinate heme iron are described in our previous report [36]. However, the primary tyrosine residues of the acceptor proteins (IsdA-N in the IsdH-N3•heme•IsdA-N system and IsdC-N in the IsdA-N•heme•IsdC-N system) were not coordinated with heme iron during MD simulations, and alternative partial charges were prepared for these residues. Charge parameters were prepared using an optimized 4-methylphenoxyde model at the B3LYP/6-31G(d) level with Gaussian 09 (Fig D in S1 File). Water molecules and charge-neutralizing ions were placed around proteins using the program Solvate 1.0 [37]. Subsequently, water molecules were added to the system with a thickness of more than 8 Å to form a box-shaped system using the LEaP module of AmberTools 13 [38]. The Amber ff99SB force field parameters [39] and the TIP3P model [40] were used for standard amino acids and water, respectively.

After construction of the initial structures, the systems were energy optimized and were then heated gradually to 300 K during 200-ps constant-*NVT* MD simulations with harmonic position restraints on the heavy atoms of solutes (force constant, 10 kcal mol^−1^ Å^−2^). During subsequent 800-ps constant-*NPT* MD simulations at 300 K and 1.0 × 10^5^ Pa, force constants of the position restraints were gradually reduced to 0 kcal mol^−1^ Å^−2^. The systems were then further equilibrated for 200 ps without position restrains, and the SHAKE algorithm [41] was employed to constrain all bond lengths involving hydrogen atoms, allowing the use of 2-fs time steps. Temperature and pressure were controlled using a Langevin thermostat [42] and a Berendsen barostat [43], respectively, and simulations were performed using the PMEMD module of AMBER12 [38].

After system equilibration, restrained MD simulations were performed for 15 ns to dock the acceptor protein with the donor complex using sets of distance restraints (Set HA; Table A in S1 File) between heme atoms of the donor NEAT•heme complex and acceptor NEAT domain atoms. As for the IsdA-N•heme•IsdC-N system, an additional restrained MD simulation was performed for 35 ns with a modified set of distance restraints (Set AC2; Table A in S1 File), and restrained MD simulations were performed using the SANDER module of AMBER 12. Subsequently, unrestrained MD simulations were performed until 1,000 ns using GROMACS version 5.0.4 [44], and the ffAmber script [45] was used to convert the AMBER topology file format to that for GROMACS. In these simulations, the P-LINCS algorithm [46] was used instead of the SHAKE algorithm, and the stochastic velocity-rescaling (V-rescale) algorithm [47–49] was used to control temperature.

### Computational details for our own *n*-layered integrated molecular orbital and molecular mechanics calculations

Additional 50-ns MD simulations were performed with two modifications for each NEAT•heme•NEAT system prior to our own *n*-layered integrated molecular orbital and molecular mechanics (ONIOM) calculations. Initially, the Oη atom of the secondary tyrosine residue of the acceptor NEAT domain was deprotonated and that of the primary tyrosine was protonated. Subsequently, a distance restraint was applied between the Oη atom of the secondary tyrosine and the iron atom with an equilibrium distance of 2.8 Å and a force constant of 1.0 kcal/mol. Initial coordinates and velocities were taken from the final (at 1,000 ns) coordinates of the unrestrained simulations described above.

QM/MM calculations were performed using a two-layer ONIOM mechanical embedding scheme with the Gaussian 09 software package. QM regions were treated with B3LYP functional using the 6-31G(d) basis set for H, C, N, and O atoms and the LANL2DZ ECP basis set [34] for iron atoms. The heme iron atom was assumed to be in a ferric high-spin state as described above. MM regions were treated with an AMBER force field, and bonds crossing the QM/MM boundaries were capped with hydrogen link atoms.

During the first 20 steps of each optimization, all atoms were allowed to move to reduce steric repulsion. Subsequently, only atoms within 7 Å of the QM region were allowed to move. To reduce the errors associated with using fixed charges in QM regions, the TAO package [50] was used to prepare initial coordinates, analyze ONIOM energies, and update MM charges. Optimization was continued until the decrease in ONIOM energy was less than 0.5 kcal/mol.

## Results and Discussion

### PES of the Fe(III)-porphine-(phenolate)_2_ complex

**Fig 2:**
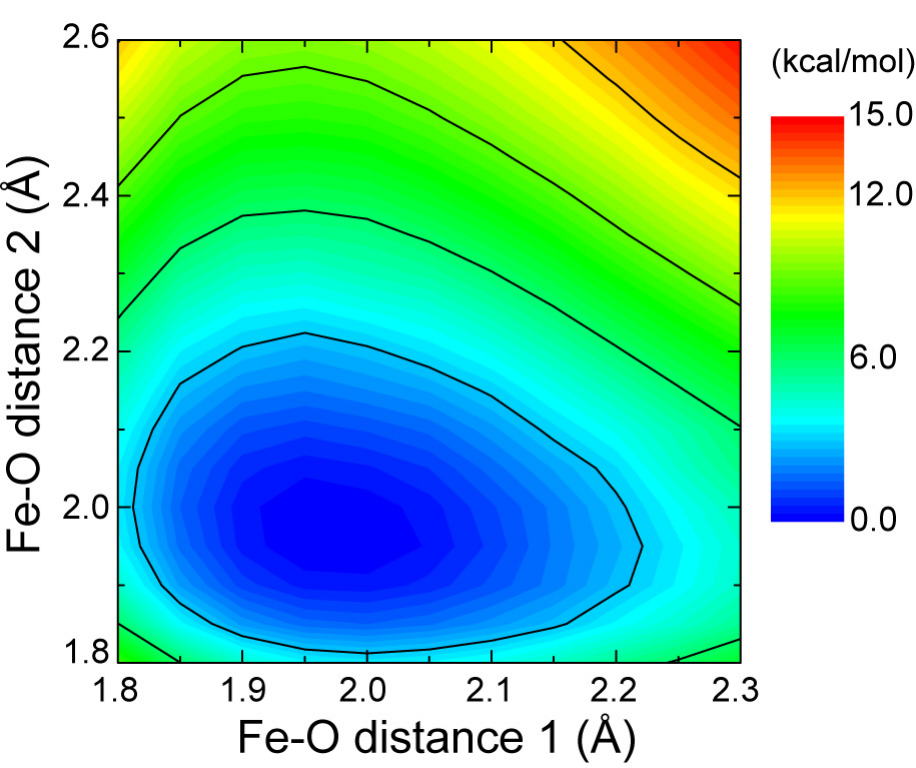
Two-dimensional potential energy surface of the system of two phenols and an Fe(III)-porphine. Black contour lines are at intervals of 3 kcal/mol.

In the “tight handclasp” model [25, 26], two tyrosine residues are coordinated to the axial positions of heme iron. To validate this structure, we calculated a relaxed PES of an Fe(III)-porphine-(phenolate)_2_ complex by changing the two Fe–O distances. The PES of this complex had a single minimum at an Fe–O distance of 1.97 Å, which was longer than the distance in the crystal structure of Fe(III)-octaethylporphinato-phenolate (1.85 Å) [51] and was also longer than that obtained for Fe(III)-porphyrin-phenolate (1.87 Å) under the same conditions (Fig 2). Thus, coordination of a phenolate anion to the iron atom is an energetically favorable reaction irrespective of the occupation of the other axial site with a phenolate anion. Therefore, the phenolate anion spontaneously coordinates with the iron atom of Fe(III)-porphine. In contrast, geometric optimization of the Fe(III)-porphine-phenolate-phenol complex failed, suggesting instability of the complex. Taken together, these observations indicate that two tyrosine residues can be simultaneously coordinated at the axial positions of the heme iron, but only if they are both deprotonated.

### Docking of two NEAT domains using MD simulation

**Fig 3:**
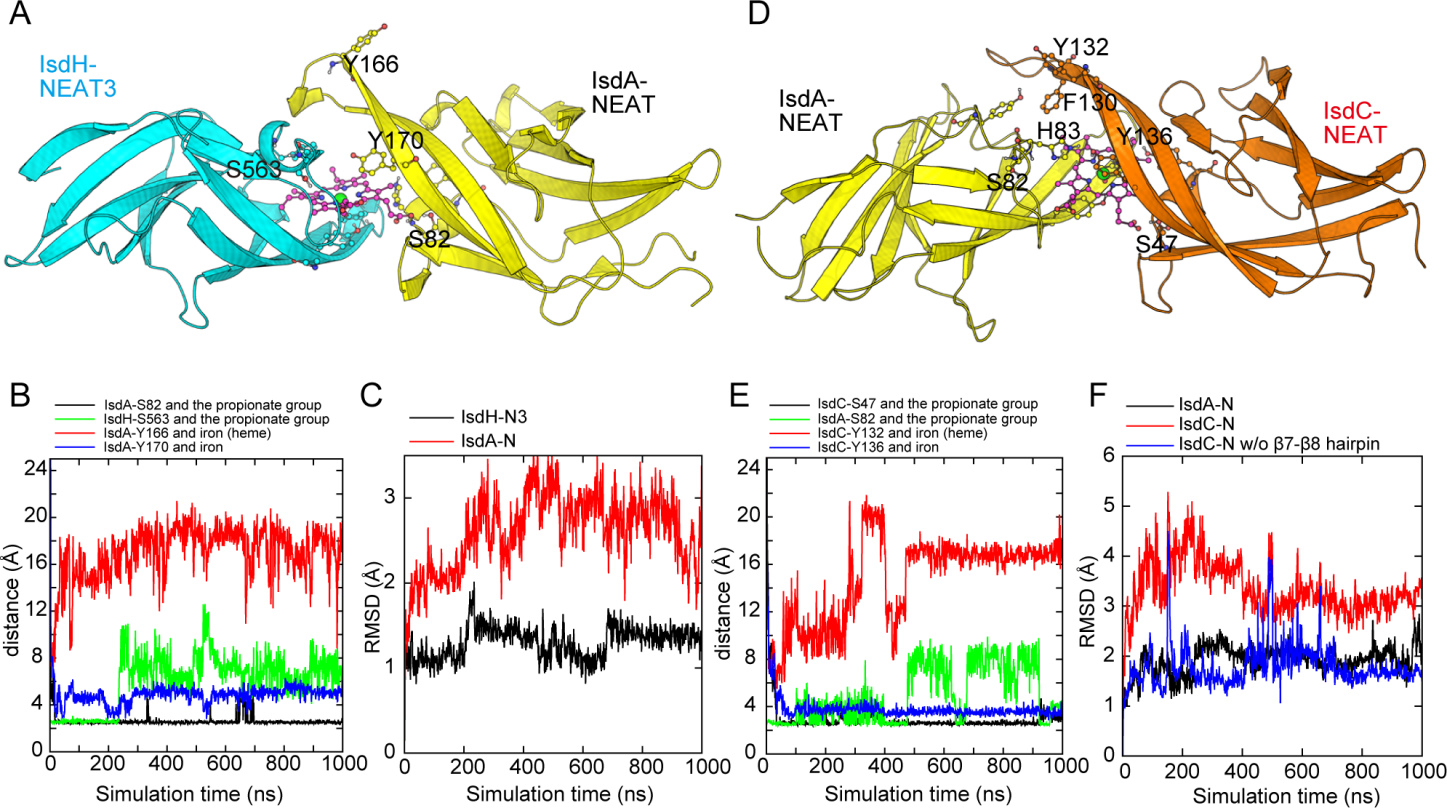
MD simulations of Isd•heme•Isd ternary complexes. (A and D) Overall structures of the docked complexes IsdH-N3, IsdA-N, IsdC-N, and heme are represented in cyan, yellow, orange, and purple, respectively. The IsdH-N3•heme•IsdA-N and IsdA-N•heme•IsdC-N snapshots were obtained in MD simulations at 1,000 and 900 ns, respectively. (B and E) Plots of distances in MD trajectories; black, green, red, and blue traces represent distances between side-chain Oγ atoms of conserved serine residues and proximal carboxyl groups of heme, between Oη atoms of the primary tyrosine residues and iron, and between the secondary tyrosine and iron, respectively. (C and F) Plots of RMSD values; crystal structures were used as reference structures. Panel F: RMSD values that were calculated excluding the β7-β8 hairpin in IsdC-N (residues Asp-118 to Tyr-136) are presented in the blue trace.

Initially, the coordination structure of the heme iron in the tight handclasp model was rationalized using the QM calculation. Subsequent docking simulations of the two NEAT domains, IsdH-N3•heme and IsdA-N or IsdA-N•heme and IsdC-N, were performed using restrained MD simulations. Distance restraints were mainly imposed between the heme iron atom and the Oη atom of the primary tyrosine residue of the acceptor protein and between the oxygen atoms of the propionate group of heme and the Oγ atoms of the serine residues (IsdH–Ser563, IsdA–Ser82, and IsdC–Ser47) that form hydrogen bonds in corresponding one-to-one NEAT•heme complexes (see Table A in S1 File for full list of the restrains). These distance restraints allowed construction of ternary complex models for both systems, with two NEAT domains arranged in two-fold pseudo symmetry around the heme molecule, although longer simulations were required for the IsdA-N•heme•IsdC-N system (Fig 3A and 3D). A hydrogen bond was formed between the propionate group of the heme that was initially exposed to the solvent and the acceptor NEAT domain (Fig 3B and 3E). Upon formation, ternary NEAT•heme•NEAT complexes were maintained for more than 900 ns in unrestrained MD simulations (S1 Video). Moreover, the structures of NEAT domains were not largely altered upon the formation of complexes, excepting for the long β7–β8 hairpin of IsdC-N, which showed large fluctuations during the MD simulation (Fig 3C and 3F).

Dissociation of the hydrogen bond between the conserved serine residue of the donor NEAT domain and the propionate group of heme was observed in all MD simulation (Fig 3B and 3E). However, the hydrogen bond with IsdA–Ser82 was maintained throughout the simulation in the IsdH-N3•heme•IsdA-N complex, but was destabilized in the IsdA-N•heme•IsdC-N complex. This observation indicates that the hydrogen bond of the propionate group of heme with the donor NEAT domain is less stable than that with the acceptor and is consistent with the results of the MD simulations for the NEAT•heme complexes: the hydrogen bond was most stable in the IsdC-N•heme complex, moderately stable in the IsdA-N•heme complex, and least stable in the IsdH-N3•heme complex [22]. This tendency is correlated with the number of negatively charged residues in the loop between strand β1b and the 3_10_-helix where the conserved serine is located [22]. Therefore, the difference in the stability of this hydrogen bond may potentially facilitate the transition of heme from donor to acceptor NEAT domains.

Plots of distances between the heme iron atom and the primary tyrosine Oη atoms of the acceptor proteins IsdA–Tyr166 and IsdC–Tyr132 indicate that coordinate bonds were not formed (Fig 3B and 3E). However, the secondary tyrosine residues of acceptor proteins approached the heme iron atom. Thus, the secondary tyrosine of the acceptor protein likely forms a coordinate bond with the heme iron in the intermediate ternary complex. Accordingly, after completion of the heme transfer reaction, the primary tyrosine is likely substituted for the secondary tyrosine, as observed in the crystal structure of the NEAT•heme complex.

### Refinement of docking models using the QM/MM method

As described above, heme iron can adopt a six-coordinate structure with two tyrosine axial ligands but only if both are deprotonated. In addition, the present MD simulations show proximity of the secondary tyrosine residue and heme iron. Hence, water molecules could enter into the interface between the NEAT domains, especially after the hydrogen bond between the conserved serine of the donor and the propionate group of heme is broken. Thus, the secondary tyrosine may be deprotonated for coordination to heme iron instead of the primary tyrosine.

To obtain equilibrium structures under this protonation condition, we performed additional 50-ns MD simulations in which primary and secondary tyrosine residues of the acceptor domain were protonated and deprotonated, respectively. These simulations indicated that a hydrogen bond was formed between protonated IsdA–Tyr166 and deprotonated IsdA–Tyr170 in the IsdH-N3•heme•IsdA-N complex. This hydrogen bond was unstable during the previous simulations, in which the primary tyrosine was deprotonated, and the secondary tyrosine was protonated (Fig 3B). On the other hand, an alternative hydrogen bond was formed between IsdA–His83 and deprotonated IsdC–Tyr136 instead of protonated IsdC–Tyr132 in the IsdA-N•heme•IsdC-N complex. These observations imply that deprotonation of the secondary tyrosine of the acceptor domain induces rearrangement of the hydrogen-bond network between the surrounding residues of heme. In addition, small movements of heme toward acceptor proteins during the previous MD simulations allowed water molecules to approach the heme, the tyrosine residues, and the IsdA–His83 of intermediate complexes.

**Fig 4:**
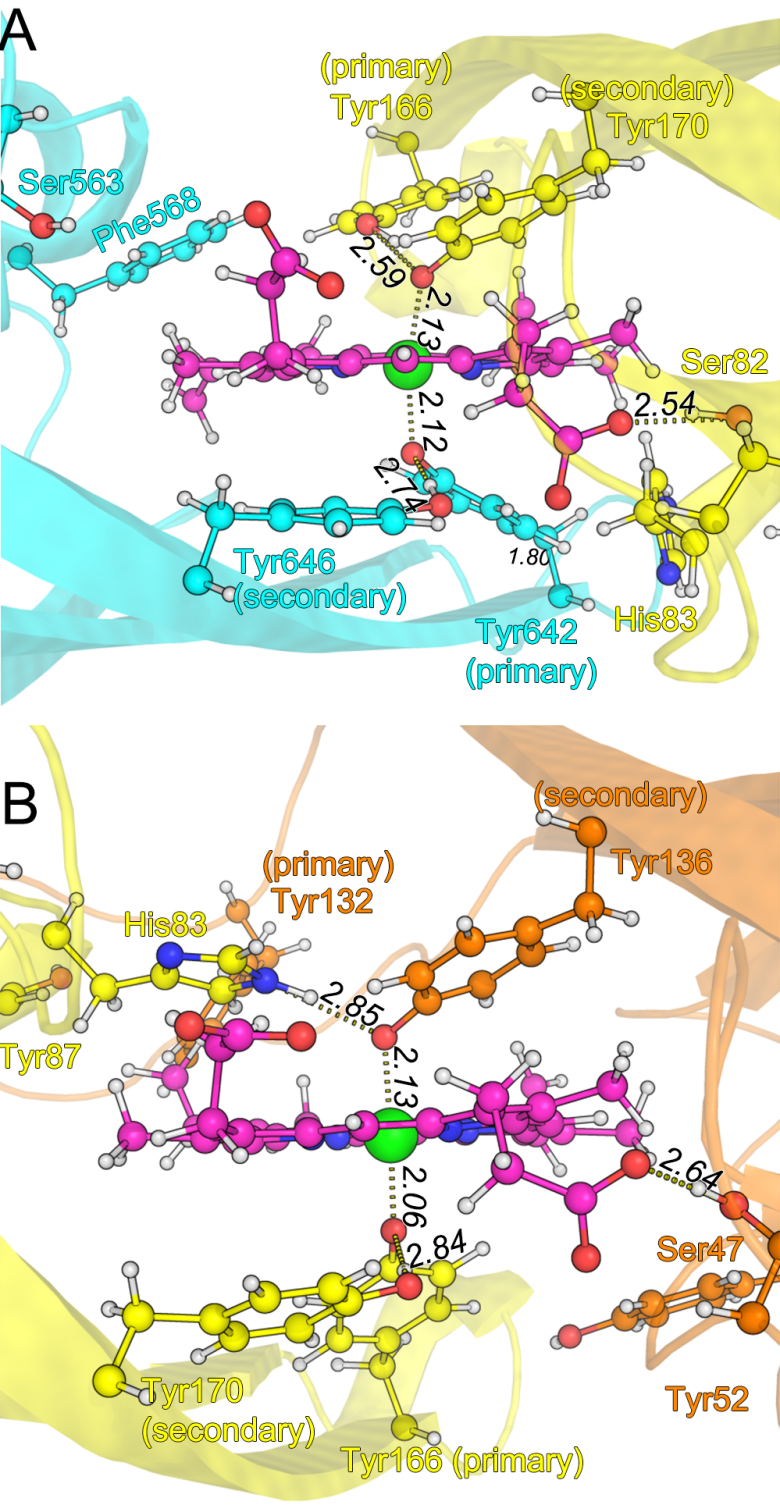
Optimization of structures using the ONIOM method. Residues of IsdH-N3, IsdA-N, and IsdC-N are shown in cyan, yellow, and orange, respectively. Heme is shown in purple. (**A**) IsdH–Tyr642 and IsdA–Tyr170 are deprotonated in the IsdH-N3•heme•IsdA-N complex and (**B**) IsdA–Tyr166 and IsdC–Tyr132 are deprotonated in the IsdA-N•heme•IsdC-N complex.

In subsequent ONIOM optimizations of the IsdH-N3•heme•IsdA-N system, heme atoms and the four tyrosine residues (primary and secondary tyrosine residues of both proteins) were included in the QM region. However, in the IsdA-N•heme•IsdC-N system, IsdA–His83 was included in the QM region, in place of IsdC–Tyr132. In both optimized structures of NEAT•heme•NEAT complexes, the Oη atoms of the primary tyrosine of the donor protein and the secondary tyrosine of the acceptor protein were located at the axial positions of the heme iron atom, with Fe–O bond lengths of 2.06–2.13 Å (Fig 4). These observations confirm that the two tyrosine residues are simultaneously coordinated with heme iron in the intermediate ternary complex. However, the Fe–O bond lengths were slightly longer than the optimal value for the Fe(III)-porphine-(phenolate)_2_ complex (1.97 Å; Fig 2). In most five-coordinated iron porphyrins, including those of one-to-one NEAT•heme complexes, the iron atom is slightly out of plane of the porphyrin ring. In the NEAT•heme•NEAT complexes, however, the iron atom was positioned in the porphyrin plane, as is typical for six-coordinated iron porphyrins. In addition, the hydrogen bonds between primary and secondary tyrosine residues in all donor domains, including those between IsdA–Tyr166 and IsdA–Tyr170 in the IsdH-N3•heme•IsdA-N complex and between IsdC–Tyr136 and IsdA–His83 in the IsdA-N•heme•IsdC-N complex, were all preserved after optimization.

PES-scan and transition-state search calculations indicated no energy barrier between reactant and product states in the coordination of heme iron with the deprotonated tyrosine of the acceptor NEAT domain and were consistent with analyses of the Fe(III)-porphine-(phenolate)_2_ complex (Fig 2). These observations indicate that the six-coordinated structure of heme iron is automatically formed when the acceptor NEAT domain with the deprotonated tyrosine residue docks to the NEAT•heme complex.

### Optimization with a protonated tyrosine

To complete the relay of heme between NEAT domains, dissociation of the Fe–O bond must occur after formation of the intermediate complex, potentially following protonation of the iron-coordinating tyrosine. Thus, further ONIOM optimizations were performed with protonation of deprotonated tyrosine residues.

**Fig 5:**
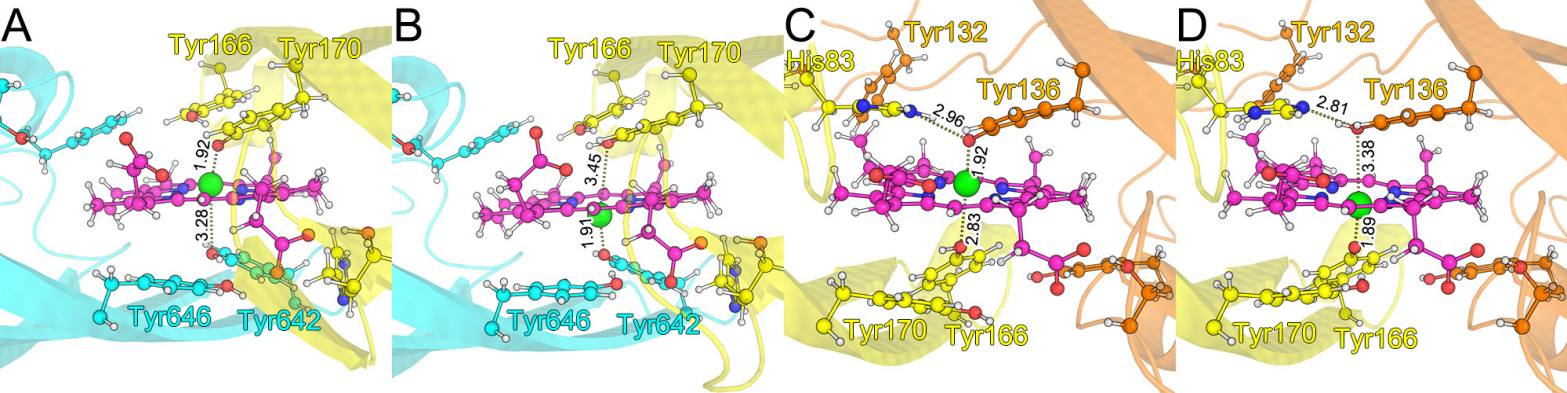
Optimized structures with an additional proton. Residues of IsdH-N3, IsdA-N, and IsdC-N are shown in cyan, yellow, and orange, respectively. Heme is shown in purple. (A) Protonated IsdH–Tyr642 and–Tyr646; (B) protonated IsdA–Tyr166 and–Tyr170 in the IsdH-N3•heme•IsdA-N system; (C) protonated IsdA–Tyr166 and–Tyr170 in the IsdA-N•heme•IsdC-N system; (D) protonated IsdA–His83 and IsdC–Tyr136; an additional proton was initially placed near the Nδ atom of IsdA–His83.

Structural optimizations were initiated with the coordinates of the intermediate ternary complexes that were optimized with deprotonated tyrosine residues in the previous section. Initially, a proton was placed near the Oη atom of IsdH–Tyr646 in the IsdH-N3•heme•IsdA-N complex. Immediately upon attachment of a proton to IsdH–Tyr646, the proton that was initially located between IsdH–Tyr642 and IsdH–Tyr646 was moved to IsdH–Tyr642 to form a protonated tyrosine, leading to dissociation of the Fe–O bond between IsdH–Tyr642 and heme (Fig 5A). Subsequently, the distance between IsdH–Tyr642 and iron grew to 3.28 Å in the optimized structure. However, the Fe–O bond with IsdA–Tyr170 became 1.92 Å, which is similar to that in five-coordinated iron porphyrin complexes. Unlike Fe(III)-porphine-phenolate-phenol complex optimizations, these ONIOM optimizations were successfully completed, presumably reflecting effective reduction of the conformational space of the tyrosine residue by the protein scaffold.

Further ONIOM optimizations were performed with protonated IsdA–Tyr170, which was the deprotonated tyrosine of the acceptor NEAT domain in the IsdH-N3•heme•IsdA-N complex. As expected, the Fe–O bond between IsdA–Tyr170 and iron was broken, with a distance of 3.45 Å, and the bond between IsdH–Tyr 642 and heme was shortened to 1.91 Å (Fig 5B).

As for the IsdA-N•heme•IsdC-N complex, we performed the ONIOM optimizations with an additional proton near the Oη atom of IsdA–Tyr170. During the subsequent optimization, the proton that was originally bound to IsdA–Tyr170 was transferred to IsdA–Tyr166 and caused dissociation of the Fe–O bond between the tyrosine residue and heme. This was similar to that observed in the IsdH-N3•heme•IsdA-N system (Fig 5C). When an additional proton was placed near the Nδ atom of IsdA–His83, in proximity to IsdC–Tyr136 instead of IsdC–Tyr132, a proton was transferred from the Nε atom of IsdA–His83 to IsdC–Tyr136 during the structural optimization, and the Fe–O bond was elongated to 3.38 Å (Fig 5D). These results suggest that dissociation of the Fe–O bond occurs in donor and acceptor domains, and that protonation of the coordinating tyrosine residue plays an important role in the transfer of heme between NEAT domains.

### Proposed mechanism of heme-transfer reaction between NEAT domains

**Fig 6:**
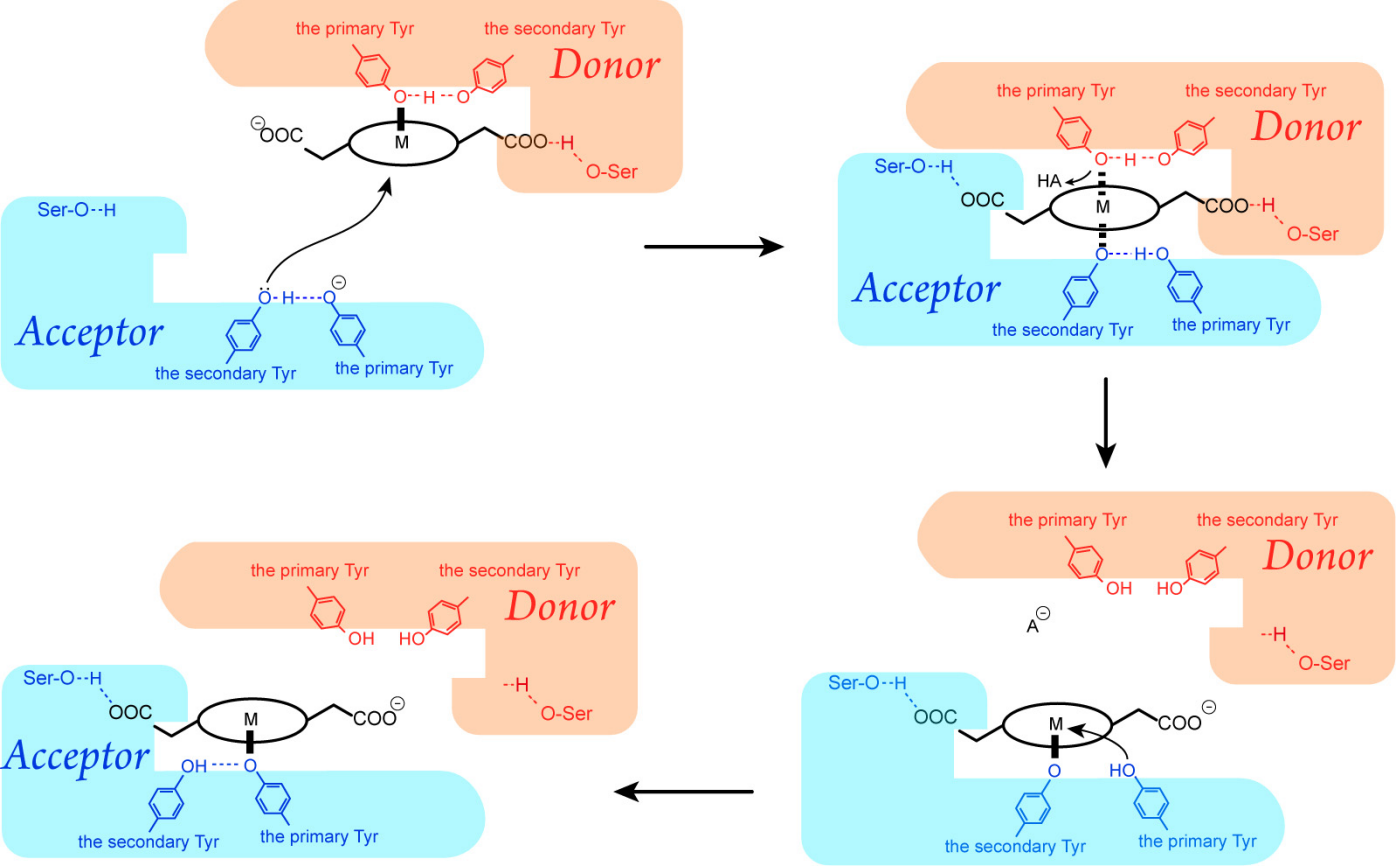
Proposed heme transfer reaction between NEAT domains. Cyan and red objects represent acceptor and donor NEAT domains, respectively. Black circles indicate porphyrin rings, and M indicates the metal chelated in the porphyrin.

Fig 6 shows the proposed reaction scheme for heme transfer between NEAT domains. The present model assumes that the secondary tyrosine of the acceptor protein is deprotonated before forming a coordinate bond with heme iron (top-left panel of Fig 6). Although the p*K*_*a*_ value of isolated tyrosine is about 10, analyses of NEAT domains [19, 22] indicate decreased p*K*_*a*_ values of primary and secondary tyrosine residues, due to the formation of a hydrogen bond between them in the apo form. Therefore, even at neutral pH, some apo Isd molecules likely carry deprotonated secondary tyrosine residues and react with the NEAT•heme complex to receive heme.

The heme-transfer reaction is viewed as an exchange of axial heme iron ligands from one side of heme to the other. Comparison with the axial ligand replacement mechanism in the Shp•heme•HtsA complex of Group A *Streptococcus* [52] provides general insight into the heme-transfer reaction between proteins. In the Shp•heme•HtsA complex, Met79 and His229 of HtsA simultaneously replace the two axial ligands of heme, Met66 and Met153, of Shp, respectively. However, this mechanism is different from ours in several aspects. Firstly, we proposed that the transfer occurs in a longitudinal direction parallel to the normal of the heme plane in the NEAT•heme•NEAT complexes, whereas in the Shp•heme•HtsA complex, it is proposed to occur in a lateral direction. Secondly, the heme iron adopts the five-coordinate structure in the NEAT•heme complexes and adopts the six-coordinate structure only in the intermediate complex. In contrast, in the Shp-HtsA system, it adopts the six-coordinate structure throughout in the one-to-one complexes and during the transfer reaction. Moreover, from a chemical point of view, the presence of a six-coordinated intermediate structure has been proposed [53, 54] based on kinetic analyses of an iron(III) tetramesitylporphyrin phenolate complex using various carboxylic acid and alcohol ligands. This exchange reaction does not change the valence and the spin state of the iron, which is consistent with ESI-MS and MCD observations of NEAT•heme complexes [24, 27–30].

The present QM/MM calculations suggest that dissociation of the Fe–O bond is triggered by protonation of the coordinating tyrosine (top-right panel of Fig 6). These calculations also suggest equal occurrence on both sides of heme in the intermediate complex. In contrast, classical MD simulations of the intermediate complex indicated that the hydrogen bond between the propionate group of heme and the donor domain was less stable than that formed with the acceptor domain, reflecting differences in heme binding affinity [14, 23, 24].

In all reported crystal structures of one-to-one NEAT•heme complexes, the Fe–O bond is formed with the primary tyrosine. Therefore, another heme transfer reaction from the secondary tyrosine to the primary tyrosine may occur in the acceptor protein (bottom-right panel of Fig 6). Thus, the present model successfully explains the heme-transfer mechanism between NEAT domains.

## Conclusion

In the present study, we modeled the structure of the intermediate NEAT•heme•NEAT complex and investigated mechanisms of rapid heme transfer between NEAT domains. QM calculations for Fe(III)-porphine-(phenolate)_2_ showed that the heme iron adopts a six-coordinated structure with two tyrosine ligands at axial positions only if both tyrosine residues are deprotonated. Accordingly, models of intermediate NEAT•heme•NEAT complex were generated in MD simulations with distance restraints between the heme iron of the donor NEAT•heme complex and the primary tyrosine residue of the acceptor NEAT domain and between the propionate group of heme and the conserved serine residue of the acceptor NEAT domain. The stability of these models was confirmed in unrestrained MD simulations that were performed for more than 900 ns. These simulations also suggest that the secondary tyrosine residue of the acceptor domain forms a coordinate bond with the heme iron instead of the primary tyrosine and that the hydrogen bond between the propionate group of heme and the donor domain may be less stable than that formed with the acceptor domain, reflecting differing heme binding affinities. In addition, the present QM/MM calculations demonstrated that the deprotonated secondary tyrosine residue of the acceptor domain and the deprotonated primary tyrosine residue of the donor domain can coordinate simultaneously with heme iron to form a six-coordinated complex. In contrast, protonated tyrosine did not coordinate with iron and resulted in a five-coordinated structure. Based on these results, we proposed a reaction scheme for heme transfer between NEAT domains (Fig 6).

**Fig 7:**
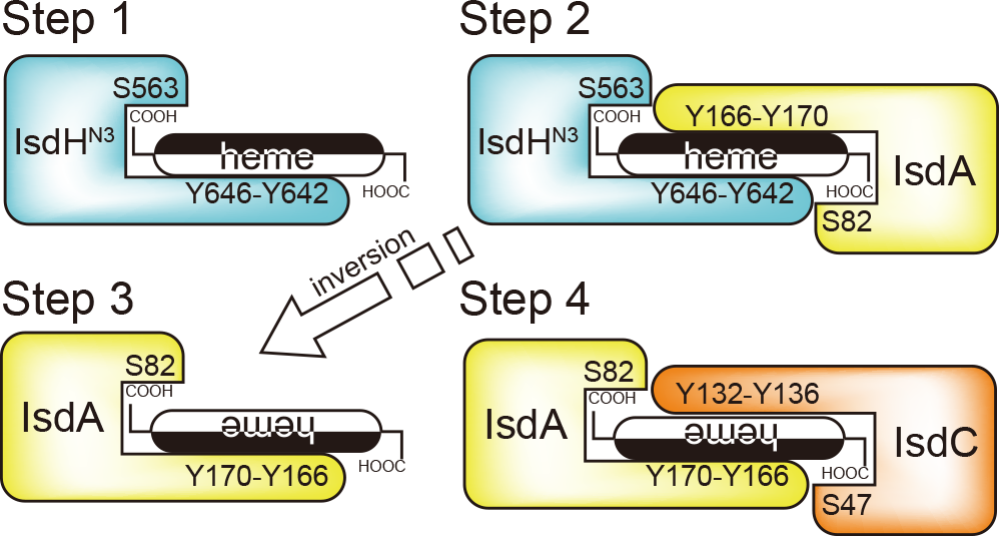
Proposed heme transfer mechanism from IsdH-N3 to IsdC via IsdA. The sides of heme are shown in black and white. Heme is inverted upon formation of the complex between acceptor and donor NEAT domains.

Fig 7 shows the entire process of heme transfer from IsdH-N3 to IsdC-N via IsdA. Similar to the intermediate structure of the S_N_2 reaction, the secondary tyrosine of the acceptor protein is on the opposite side of the primary tyrosine of the donor protein. In particular, the secondary tyrosine of the acceptor protein corresponds to the nucleophile, and the primary tyrosine of the donor protein corresponds to the leaving group. Because the stereochemistry is inverted after the S_N_2 reaction, the side of heme on which the tyrosine binds changes after each heme transfer reaction. Accordingly, IsdA-N binds the side of heme opposite to that of IsdH-N3 (Fig 7; Steps 2 and 3), as illustrated in crystal structures of the IsdA-N•heme complex showing that IsdA-N can bind at both the sides of heme (Fig E in S1 File).

In recent studies, similar iron-uptake systems were successively characterized in human pathogens such as *Listeria monocytogenes* [55, 56], *S*. *lugdunensis* [57], *B*. *cereus*, and *B*. *anthracis*. Hence, the present model may be used in further studies on heme transfer mechanisms in these pathogens and may facilitate the development of therapeutic inhibitors of bacterial iron uptake.

## Supporting Information

S1 File Supporting Figs and table. An outline view of the Isd system of *Staphylococcus aureus* (**Fig A**). A model of Fe(III)-porphine and two phenolate molecules for PES scan (**Fig B**). Initial coordinates for MD simulations (**Fig C**). Distance restraints used for MD simulations (**Table A**). Partial charges used for tyrosinate residues in the apo-form of Isd-NEAT domains (**Fig D**). Differences in heme positions on IsdA (**Fig E**).
(PDF)

S1 Video 1000-ns IsdA-N•heme•IsdC-N MD simulations. (MP4)
