# Supplementary material for: Rapid Heme Transfer Reactions between NEAr Transporter Domains of Staphylococcus aureus: A Theoretical Study Using QM/MM and MD Simulations
Source: PLoS One. 2015 Dec 14;10(12):e0145125. doi: 10.1371/journal.pone.0145125 (PMC4684392; doi:10.1371/journal.pone.0145125)
Supplement: S1 File — An outline view of the Isd system of Staphylococcus aureus (Fig A). A model of Fe(III)-porphine and two phenolate molecules for PES scan (Fig B). Initial coordinates for MD simulations (Fig C). Distance restraints used for MD simulations (Table A). Partial charges used for tyrosinate residues in the apo-form of Isd-NEAT domains (Fig D). Differences in heme positions on IsdA (Fig E). (PDF) [file pone.0145125.s001.pdf]

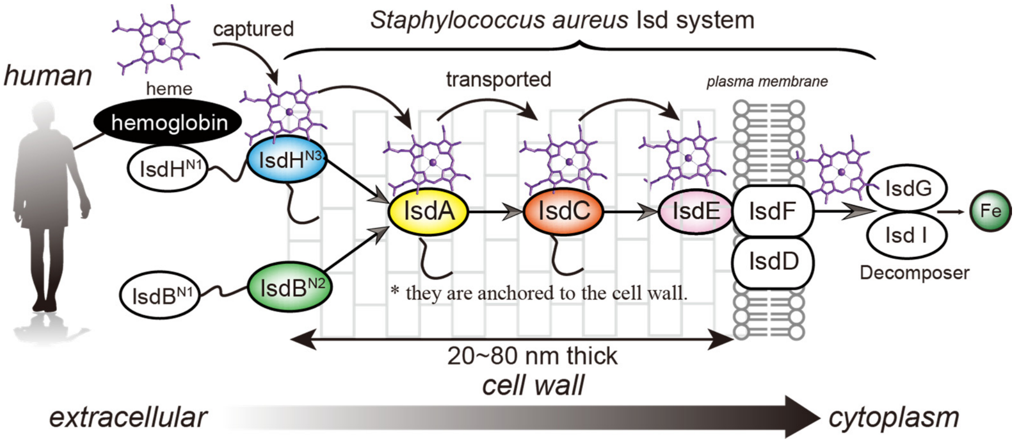

Figure A. An outline view of the Isd system of *Staphylococcus aureus*. Hemoglobin is initially sequestered by IsdH-N1/N2 (or IsdB-N1), and the extracted heme is subsequently bound to IsdH-N3 (or IsdB-N2). Heme is then transferred across the cell wall for decomposition by IsdG/IsdI.

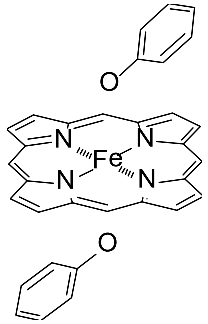

Figure B. A model of Fe(III)-porphine and two phenolate molecules for PES scan.

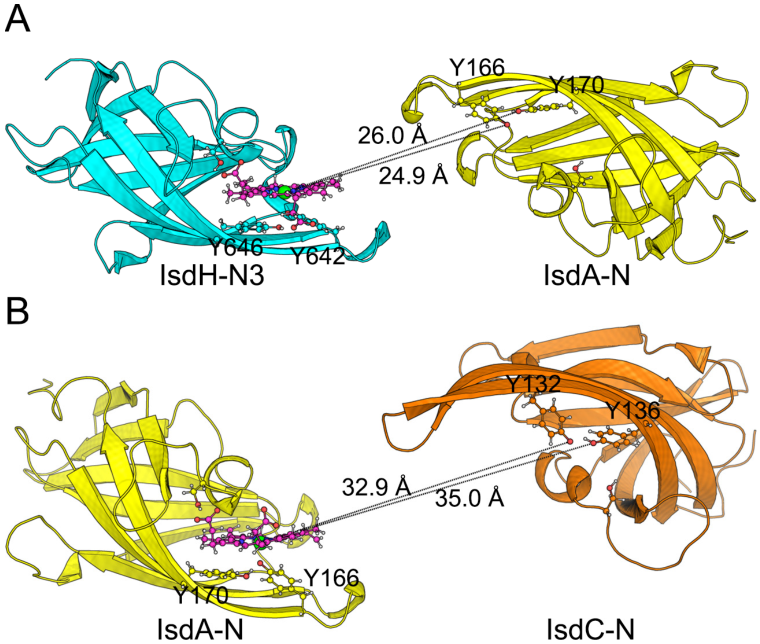

Figure C. Initial coordinates for MD simulations. (**A**) An IsdH-N3–heme complex with IsdA–N. (**B**) An IsdA–N–heme complex with IsdC–N.

Table A. Distance restraints used for MD simulations[a]

	Atom 1	Atom 2	Equilibrium distance r (Å)	Force constant k (kcal mol^{-1} Å^{-2})
IsdH-N3•heme•IsdA-N (Set HA)	IsdH^{N3}-Ser563-OG	Heme-O1D	2.8727	10.0
	IsdH^{N3}-Tyr646-OH	Heme-FE	4.0461	30.0
	IsdA-Tyr166-OH	IsdA-Tyr170-OH	2.700	0.1
	IsdA-Tyr166-OH	Heme-FE	2.200	0.1
	IsdA-Ser82-OG	Heme-O1A	2.8727	0.1
	Heme-HVT4	IsdA-Trp113-CA	3.000	0.1
IsdA-N•heme•IsdC-N (set AC1)	IsdA-Ser82-OG	Heme-O1D	2.8727	10.0
	IsdA-Tyr170-OH	Heme-FE	4.0461	10.0
	IsdC-Tyr132-OH	IsdC-Tyr136-OH	2.507	1.0
	IsdC-Tyr132-OH	Heme-FE	2.200	0.1
	IsdC-Ser47-OG	Heme-O1A	2.8727	0.1
	Heme-HVT2	IsdC-Trp77-CA	3.000	0.1
IsdA-N•heme•IsdC-N (set AC2)	IsdA-Ser82-OG	Heme-O1D	2.8727	1.0
	IsdA-Tyr170-OH	Heme-FE	4.0461	1.0
	IsdC-Tyr132-OH	IsdC-Tyr136-OH	2.507	0.1
	IsdC-Ser47-OG	Heme-O1A	2.8727	0.05

[a]Atom names are presented in Figure . Forces acting on each object were calculated as $k(R-r)^2$, where R is the distance between objects (Å), k is the force constant (kcal mol^{-1} Å^{-2}), and r is the equilibrium distance (Å).

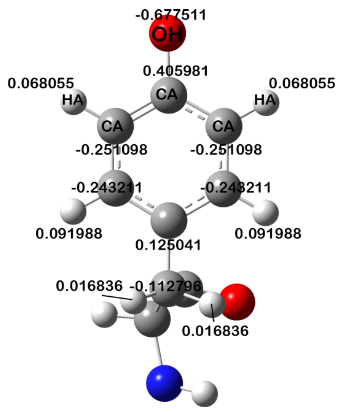

Figure D. Partial charges used for tyrosinate residues in the apo-form of Isd-NEAT domains. Partial changes were calculated using the restrained electrostatic potential (RESP) method with the ANTECHAMBER module in AmberTools 13.

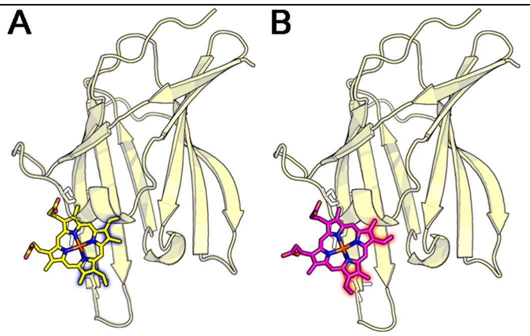

Figure E. Differences in heme positions on IsdA. Both sides of heme can enter the binding site of IsdA (PDB: 2ITF). (**A**) and (**B**) represent A and B positions of heme in the A chain, respectively.
